# Desorption Electrospray Ionization Cyclic Ion Mobility-Mass Spectrometry Imaging for Traumatic Brain Injury Spatial Metabolomics

**DOI:** 10.1021/acs.analchem.4c02394

**Published:** 2024-08-06

**Authors:** Dmitry Leontyev, Hernando Olivos, Bindesh Shrestha, Pooja M. Datta Roy, Michelle C. LaPlaca, Facundo M. Fernández

**Affiliations:** † School of Chemistry and Biochemistry, Georgia Institute of Technology, Atlanta, Georgia 30332, United State; ‡ Waters Corporation, Milford, Massachusetts 01757, United State; § Coulter Department of Biomedical Engineering, Georgia Institute of Technology/Emory University, Atlanta, Georgia 30332, United State; ∥ Parker H. Petit Institute for Bioengineering and Bioscience, Atlanta, Georgia 30332, United States

## Abstract

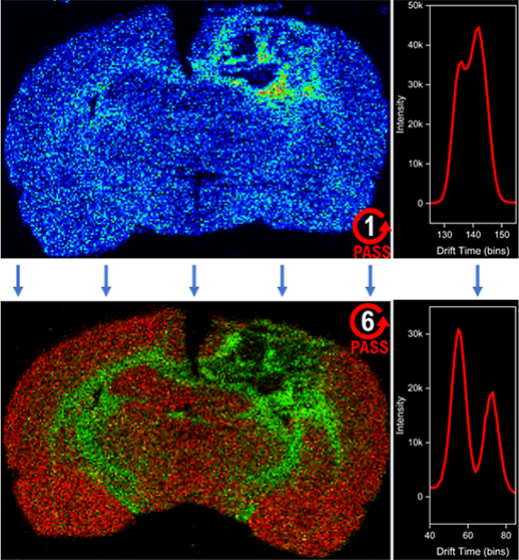

Lipidomics focuses on investigating alterations in a wide variety of lipids that harness important information on metabolic processes and disease pathology. However, the vast structural diversity of lipids and the presence of isobaric and isomeric species creates serious challenges in feature identification, particularly in mass spectrometry imaging experiments that lack front-end separations. Ion mobility has emerged as a potential solution to address some of these challenges and is increasingly being utilized as part of mass spectrometry imaging platforms. Here, we present the results of a pilot mass spectrometry imaging study on rat brains subjected to traumatic brain injury (TBI) to evaluate the depth and quality of the information yielded by desorption electrospray ionization cyclic ion mobility mass spectrometry (DESI cIM MSI). Imaging data were collected with one and six passes through the cIM cell. Increasing the number of passes increased the ion mobility resolving power and the resolution of isobaric lipids, enabling the creation of more specific maps. Interestingly, drift time data enabled the recognition of multiply charged phosphoinositide species in the complex data set generated. These species have not been previously reported in TBI MSI studies and were found to decrease in the hippocampus region following injury. These changes were attributed to increased enzymatic activity after TBI, releasing arachidonic acid that is converted to eicosanoids to control inflammation. A substantial reduction in NAD and alterations in other adenine metabolites were also observed, supporting the hypothesis that energy metabolism in the brain is severely disrupted in TBI.

## Introduction

Mass spectrometry imaging (MSI) is a powerful tool to generate ion maps for a wide variety of metabolites in situ without the need of specific labels. Visualization of metabolite changes in tissue regions of interest (ROI) contributes to the study of poorly understood biological processes and can add information to complementary modalities of spatial ‘omics experiments and other imaging techniques.^1^ Among its many applications, MSI has been leveraged to study metabolic disorders in tissue^2,3^ and to better understand disease pathology.^4−6^

Imperative to understanding any biological alterations in living systems is to determine the structural identity of the metabolites being imaged in as much detail as possible. In contrast to liquid chromatography mass spectrometry (LC–MS), MSI is traditionally conducted without front-end separations, therefore complicating feature annotation. Limited dynamic range, spectral overlaps with matrix compounds, and insufficient metabolite coverage are some of the hurdles that limit the detection of lower abundance metabolites in MSI.^7^ Feature annotation becomes even more challenging when attempting to increase the level of structural detail for isobaric species. This is particularly true for lipids, as there are an estimated 150,000 unique lipid compounds,^8^ many of which are isomeric and difficult or even impossible to distinguish by MSI alone. While most MSI experiments can readily determine lipid sum composition (e.g., PC(34:1)), such compositions typically comprise many different isomers that vary in length and position of the fatty acyl chains, and/or the location of C–C double bonds.^8^ Because differences in lipid structure can lead to significant differences in biological activity,^8^ it is desirable to distinguish isobaric and isomeric lipids. However, despite marked progress in the field, achieving this level of structural specificity in MSI is still a considerable analytical challenge. To help address these limitations, MSI instrumentation has been increasingly equipped with ion mobility separation stages and a plethora of complementary ionization front ends that expand metabolite coverage and improve specificity, leading to more meaningful and translatable biological findings.^9−11^

In ion mobility (IM) spectrometry, gas-phase ions travel through a buffer gas under the influence of a weak electric field with varying frequencies of ion-gas collisions that depend on the ions’ collision cross section (CCS) values.^12^ IM separations occur in the millisecond time scale, making them suitable for nesting with time-of-flight (TOF)-based MS platforms.^13^ The two types of dispersive IM technologies more commonly employed in MSI are traveling wave IM^14^ (TWIM) and trapped IM spectrometry^15,16^ (TIMS). In TWIM, radio frequency and direct current voltages are periodically applied to a stacked-ring ion guide yielding electric field waves that drive the movement and separation of ions.^17^ To increase TWIM resolving power, a cyclic TWIM IM (cIM) cell that can subject ions to multiple IM passes was first reported in 2019, effectively increasing resolution via its increased path length.^18^ In a cIM experiment, IM resolving power increases proportionally to the square root of the number of passes through the cell, therefore offering great experimental flexibility to resolve specific isobar pairs. TIMS, another powerful technique used in MSI, relies on the release of ions as they are entrained by the gas flow within a dual ion funnel, while simultaneously being repelled by an electric field that traps such ions in place until the field strength is sufficiently decreased, selectively releasing ions according to their CCS.^19^

IM-enabled MSI allows isobars and isomers to be resolved and individually imaged in many cases.^15,16,20−27^ However, separating most lipid isomers requires IM resolving powers of 250 or greater,^23^ and has only been demonstrated by a handful of research groups, primarily employing high resolution TIMS.^15,24−27^ Other approaches to image lipid isomers involving chemical derivatization and MS^*n*^ have been reported, but they typically operate in a more targeted fashion^28−31^ and are therefore not suitable for discovery-type experiments. IM-enabled MSI can also be leveraged to perform trend line analysis to separate compound types in drift time (*t*_d_) vs. *m*/*z* plots, aiding in MSI metabolite annotation.^32,33^ Another major advantage of coupling IM to MSI is that the IM separation stage can effectively filter out background noise,^15,33−35^ reducing unnecessary spectral complexity and increasing signal-to-noise ratios.^24,33^

In this study, we describe the first evaluation of desorption electrospray ionization^36^ (DESI) cIM MSI to conduct tissue spatial metabolomics studies in rat brains following traumatic brain injury (TBI). TBI is a complex condition caused by a physical blow to the head, altering normal brain function and causing long-term physical, emotional, and cognitive disabilities.^37,38^ Despite the 2.5–3 million TBI incidents per year in the US alone,^39^ TBI pathology is not fully understood and there is a lack of reliable diagnostic and prognostic tools.^40^ A number of MSI studies have focused on TBI,^5,6,41−50^ but none have evaluated DESI cIM MS in such context. Results presented here show significant alterations in the hippocampus for lipids and various low mass metabolites, such as nucleotides, amino acids, and peptides following TBI. We also show that cIM enables the resolution and imaging of individual lipid isobars, producing highly specific molecular images. Moreover, IM increases metabolite annotation confidence by readily separating groups of multiply charged ions from singly charged ones, simplifying MS image analysis.

## Materials & Methods

### Chemicals and Materials

Isopentane (≥99%; Sigma-Aldrich, St. Louis, MO) was used to prepare dry ice baths to snap freeze brains. Deionized water was used to ice-mount brain tissues to the microtome specimen holder. Brain sections were collected onto Superfrost Plus Microscope Slides (Fisherbrand). The DESI solvent mixture was prepared using water (Milli-Q, 18.2 MΩ cm, less than 5 ppb total organic carbon), methanol (Fisher Optima, LC MS grade) and leucine-enkephalin (>95%, Sigma-Aldrich L9133). l-α-phosphatidylinositol-4-phosphate standards (Brain, Porcine, 840045) and l-α-phosphatidylinositol-4,5-bisphosphate standards (Brain, Porcine, 840046) were purchased from Avanti Lipids (Birmingham, AL).

### Animals and Controlled Cortical Impact Procedures

All animal procedures were conducted in accordance with the guidelines set forth in the Guide for the Care and Use of Laboratory Animals (U.S. Department of Health and Human Services, Washington, DC, USA, Pub no. 85-23, 1985) and approved by the Georgia Institute of Technology Institutional Animal Care and Use Committee (protocol #A100188). To induce open head TBI, male Sprague–Dawley rats (Charles River, Wilmington, MA, USA) weighing between 300 and 400 g were kept under 12 h reverse light–dark cycles with food and water given ad libitum. Rats were randomly assigned to the sham group (*n* = 4) or the injury group (*n* = 4). The craniectomy and controlled cortical impact procedures are detailed in the Supporting Information section. Three days post injury, animals were transcardially perfused with cold 0.1 M phosphate buffer (pH 7.4).

### Tissue Slide Preparation

Brain tissues were carefully extracted and snap frozen in a dry ice-cooled isopentane bath and stored at −80 °C until sectioning. Brains were ice mounted with deionized water and coronally sectioned to 12 μm, −2.5 to −3.5 mm relative to bregma using a Thermo Shandon NX70 Cryostar cryostat (Waltham, MA). Sham and TBI rat brains were sectioned in pairs with a sham section at the top of the slide and an injured section at the bottom, producing four different sets of slides. The slides were then stored at −80 °C until MS imaging.

### Mass Spectrometry, Imaging, and Ion Mobility Parameters

Slides were placed in a vacuum desiccator 10 min prior to imaging. Imaging data were collected on a SELECT SERIES Cyclic IMS (Waters Corporation, Milford, MA) instrument equipped with a DESI-XS ion source (Waters Corporation, Milford, MA). Slides from all four rat pairs were imaged in positive and negative ion modes using one pass through the cyclic mobility cell. Slides from one pair of rat brain sections were selected to be imaged in positive and negative ion modes using a six-pass method. Images were collected from *m*/*z* 50 to 1200 with a 70 μm raster width at a scan rate of 350 μm s^–1^ (0.2 s per pixel). The six-pass method was optimized for *m*/*z* 734 to *m*/*z* 870 in positive and negative ion modes. The DESI solvent mixture (methanol/water, 98:2, with leucine-enkephalin, 200 pg ul^–1^) was electrosprayed at 0.7 kV with a 2 μL min^–1^ flow rate using a nanoflow pump (M-Class uBSM, Waters) and gas pressure of 15 psi. Leucine-enkephalin was used for lock mass correction in positive and negative ion modes. Additional MS settings are provided as a supplementary.txt file. MS2 data was collected for confirming phosphoinositide annotations using the DESI settings noted above, with a 15 LM resolution and 20 V Transfer CE.

### Image Processing and Data Analysis

MS images were processed with the Waters HDI 1.6 software using leucine-enkephalin as the lock mass (*m*/*z* 554.2615 for negative ion mode and 556.2771 for positive ion mode), 0.25 Da lock mass tolerance, 500 min signal intensity, 30 min sample frequency, 10 s sample duration, 0.05 Da *m*/*z* window, 40,000 MS resolution, 1 bin start drift, 200 bins stop drift, 2 bins drift window and 4 bins HD min peak width. Drift time data of these HDI-processed files were reported in bins. Averaged *m*/*z* and *t*_d_ lists (target lists) containing the 1000 most abundant features for positive and negative ion mode single-pass data were built in HDI using the four sets of rat brains (*n* = 8 total), a 0.03 Da *m*/*z* tolerance and 0.3 bin drift tolerance. Global lock mass correction with leucine-enkephalin was applied to the six pass data in MassLynx to obtain accurate *m*/*z* values. Metabolite abundance data were extracted from the hippocampus contralateral to the craniectomy for all eight rats using the polygon tool in HDI. The built-in HDI image correlation filter was used to find any additional ion distributions that might be relevant to injury. The coelution filter was used with a 0.002 Da window and a 200 bin *t*_d_ tolerance to search for isobars. Arrival time distribution plots and *t*_d_ plots were created in Origin after exporting *t*_d_ data from Waters MassLynx and HDI, respectively. Ion image color scales were manually adjusted in HDI to enhance the perceived image contrast. H&E images were adjusted to improve brightness and contrast.

### Feature Annotation

MSI putative feature annotation was based on matches to *m*/*z* values from the averaged target lists and assisted by images from previously annotated ions. LIPID MAPS^51^ (https://www.lipidmaps.org) was used to putatively annotate the majority of lipids, while HMDB (https://hmdb.ca) was used to putatively annotate most nonlipid low molecular weight species. The *m*/*z* tolerances used for LIPID MAPS and HMDB were ±0.005 Da and ±5 ppm, respectively. Most annotations had a mass error ranging between 1 and 3 ppm; those greater than 3 ppm were rarely considered. The adduct ions searched for in positive ion mode were [M + H]^+^, [M + Na]^+^, [M + K]^+^ and [M + 2H]^2+^. The adduct ions searched for in negative ion mode were [M – H]^−^, [M – 2H]^2–^ and [M – 3H]^3–^. Liquid chromatography mass spectrometry experiments were conducted to assign annotations with better confidence, as described in the Supporting Information section.

## Results & Discussion

### Single Pass Data Trendline Analysis

**Figure 1:**
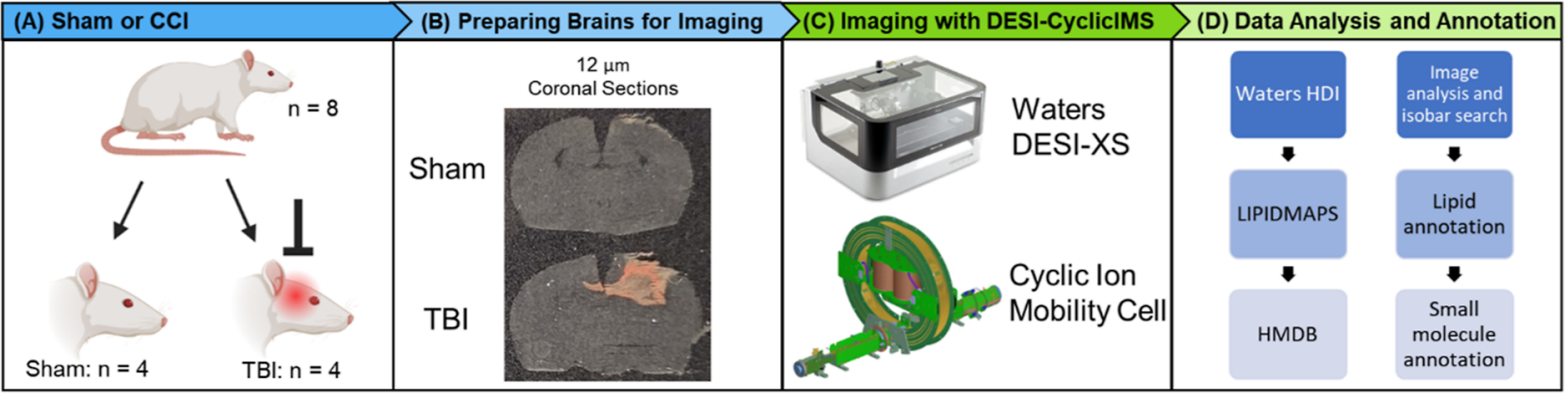
Study workflow. (A) Male Sprague–Dawley rats (*n* = 8) were divided into a sham group (*n* = 4) and a TBI (*n* = 4) group. (B) Rats were sacrificed 72 h post injury, brains were extracted, flash frozen, and coronally sectioned at 12 μm. Sham and TBI rat brains were sectioned in pairs with a sham section at the top of the slide and a TBI section at the bottom, producing four different sets of slides. (C) Imaging data were collected on a Waters Select Series Cyclic IMS platform equipped with a Waters DESI-XS ion source. Slides from all four rat pairs were imaged in positive and negative ion modes using one pass through the cyclic mobility cell. Slides from one rat pair were also imaged in positive and negative ion modes using a six-pass method. (D) Data were processed and analyzed in Waters HDI 1.6. LIPID MAPS was used to putatively annotate lipids and HMDB was used to annotate other small molecules.

The experimental workflow followed in this study is summarized in Figure 1. Drift time (*t*_d_) plots from one-pass data were examined to identify clusters of ions that deviated from the singly protonated or singly deprotonated trendlines, particularly those with lower t_d_ than average (Figure S1). Lower *t*_d_ were attributed to multiply charged ions, different biomolecular classes and various adducts. In negative ion mode, several multiply charged lipid ion groups were identified, including [M – 2H]^2–^ phosphoinositides (PIP), cardiolipins (CL), gangliosides, and [M – 3H]^3–^ gangliosides. Singly deprotonated species that deviated from the [M – H]^−^ lipid trendline included ribonucleosides, ribonucleotides, glycans and peptides. These ions had slightly lower *t*_d_ than the lipids but were scattered and did not cluster as well as multiply charged ions. In positive ion mode one clear [M + 2H]^2+^ CL cluster was observed. The only other ion class that deviated from the [M + H]^+^ lipid trend line were the [M + Na]^+^ and [M + K]^+^ lipids but did not cluster well. Overall, *t*_d_ vs *m*/*z* plots were found to be particularly useful in negative ion mode to group multiply charged ions and biomolecules other than lipids but had less utility in positive ion mode since far less species were accurately grouped by chemical class.

**Figure 2:**
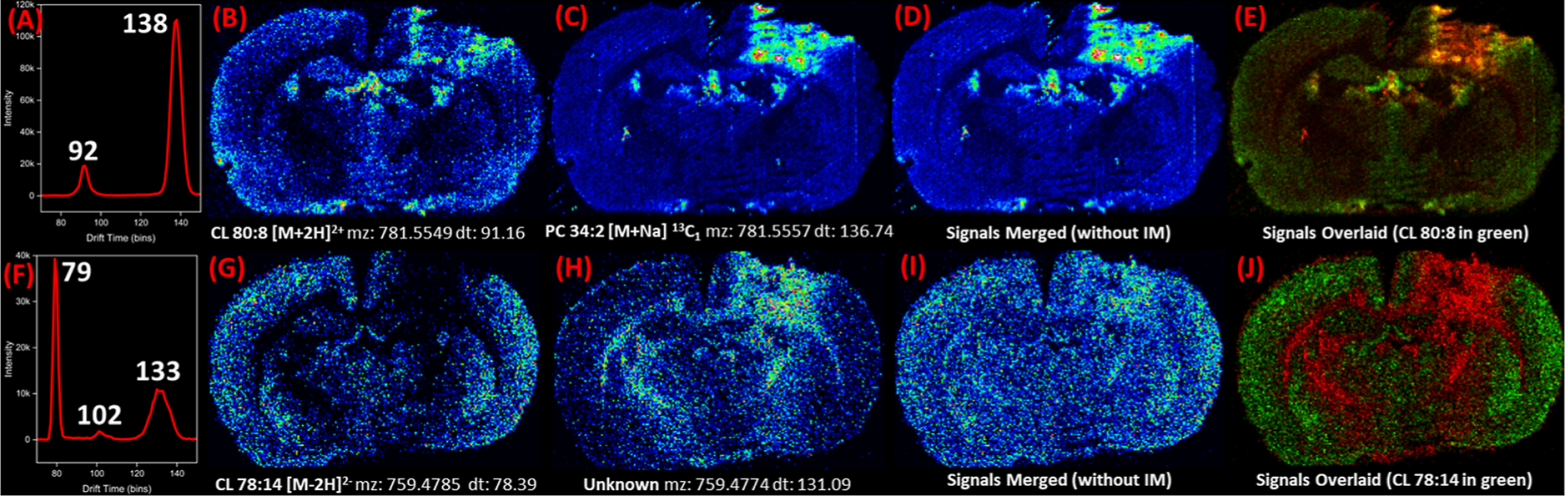
Separating doubly charged cardiolipins from singly charged isobars with one pass cyclic IM experiments. The top row of images shows separation of CL(80:8) [M + 2H]^2+^ from the isobaric PC(34:2) [M + Na] ^13^C_1_ isotope. The bottom row of images shows separation of CL(78:14) [M – 2H]^2–^ from an isobaric unknown lipid. (A) Arrival time distribution trace showing the weaker CL(80:8) [M + 2H]^2+^ ion signal at a *t*_d_ of 92 bins being separated from the stronger isobaric PC(34:2) [M + Na] ^13^C_1_ isotope signal at 138 bins. (B) Ion image of CL(80:8) [M + 2H]^2+^. (C) Ion image of the PC(34:2) [M + Na] ^13^C_1_ isotope. (D) Merged ion image of CL(80:8) [M + 2H]^2+^ and the PC(34:2) [M + Na] ^13^C_1_ isotope, showing that without IM the weaker signal would be overpowered by the PC(34:2) [M + Na] ^13^C_1_ isotope. (E) Overlaid ion images, with the CL in green and the PC signal in red, showing differences in the distribution of the two isobaric signals. (F) Arrival time distribution trace showing the CL(78:14) [M – 2H]^2–^ signal at a *t*_d_ of 79 bins being separated from unknown isobaric signals at *t*_d_ of 102 and 133 bins. (G) Ion image of CL(78:14) [M – 2H]^2–^ distributed throughout the gray matter and missing from the injury site (H) Ion image of an unknown lipid at a *t*_d_ of 133 bins. This ion was distributed throughout the white matter and lesion. (I) Ion image of CL (78:14) [M – 2H]^2–^ and the unknown lipid at 133 bins merged, showing that without IM the cardiolipin signal is obscured by the interfering isobaric signal. (J) Ion images overlaid with the CL signal in green and the unknown lipid signal at 133 bins in red, showing that these isobaric signals had opposite (i.e., spatially complementary) distributions. See Figure S2 for H&E images of rat 3.

Singly charged ions isobaric to doubly charged species were investigated in detail, as these are common spectral interferences that can skew MSI experiments. Several doubly charged CL separating in the IM dimension from isobaric (±0.002 Da) lipids were detected in positive and negative ion modes (Figure 2). In positive ion mode, the interfering isobaric species were isotopes of high intensity sodium or potassium lipid adducts that masked the true spatial distribution of CL. Figure 2A shows the cIM arrival time distribution trace at *m*/*z* 781.55 with a signal at a *t*_d_ of 92 bins corresponding to CL(80:8) [M + 2H]^2+^ and a separate signal at 138 bins corresponding to the PC(34:2) [M + Na] ^13^C_1_ isotope. The lower t_d_ signal was attributed to CL(80:8) as the [M + 2H]^2+^ was expected to have a lower *t*_d_ than a singly charged lipid at an almost identical *m*/*z*. The PC(34:2) [M + Na] ^13^C_1_ signal overpowered that of CL(80:8) such that when the signals were merged, as if no IM separation was employed, the ion image resembled that of PC(34:2) [M + Na]^+^, particularly in the injury region (top right of Figure 2B,D). In negative ion mode, two isobaric ions were found to have starkly different spatial distributions than CL. The arrival time distribution plot at *m*/*z* 759.47 comprised a peak at 79 bins corresponding to CL(78:14) [M – 2H]^2–^ and two isobar signals at 102 and 133 bins. When the signals for the CL and the 133-bin isobar were overlaid, it was observed that they had spatially complementary distributions (Figure 2J). CL(78:14) was distributed throughout gray matter structures outside the lesion area, while the isobar was distributed throughout the white matter and the lesion. When both signals were merged, the white matter and lesion areas were filled with the isobaric signal, masking the true distribution of CL(78:14) (Figure 2G,I). Figure S2 shows H&E images of this brain (rat 3) that highlight the impact area and lesion. IM separation prior to mass analysis allowed for the separation of isobaric ions that had starkly different spatial distributions from one another. These isobaric ions would require a fwhm mass resolution of almost 700,000 at *m*/*z* 759.4785 to be resolved by MS alone, a figure of merit only achievable through high end FT mass analyzers. Additional examples of this type of separations are provided in Figure S3.

### Six-Pass Data

Subjecting ions to six passes through the cyclic IM cell increases the effective ion path length, in turn increasing IM resolving power and resolution. With this increased separation power, ions with closely related structures can be more easily resolved, leading to more specific metabolite detection. One pass through the IM cell yielded a resolving power of ∼65 (*t*_d_/Δ*t*_d_), whereas six passes resulted in ∼140. Increasing the number of passes beyond this number also increases the possibility of higher mobility ions catching up to slower ones, known as a wraparound effect. For targeted experiments, wraparound is mitigated by the use of IMS^*n*^ approaches that only subject a thin slice of the ion packet to a second mobility separation after ejection of any potentially interfering ions. While the six-pass resolving power does not reach the 250 value estimated to fully resolve most lipid isomers, the increased resolution still separates many isobaric lipids that would be otherwise unresolved in one pass experiments due to their structural similarity. The acquisition time for a pair of brains using the 6-pass method was approximately 8.5 h whereas the 1-pass method was 6 h.

**Figure 3:**
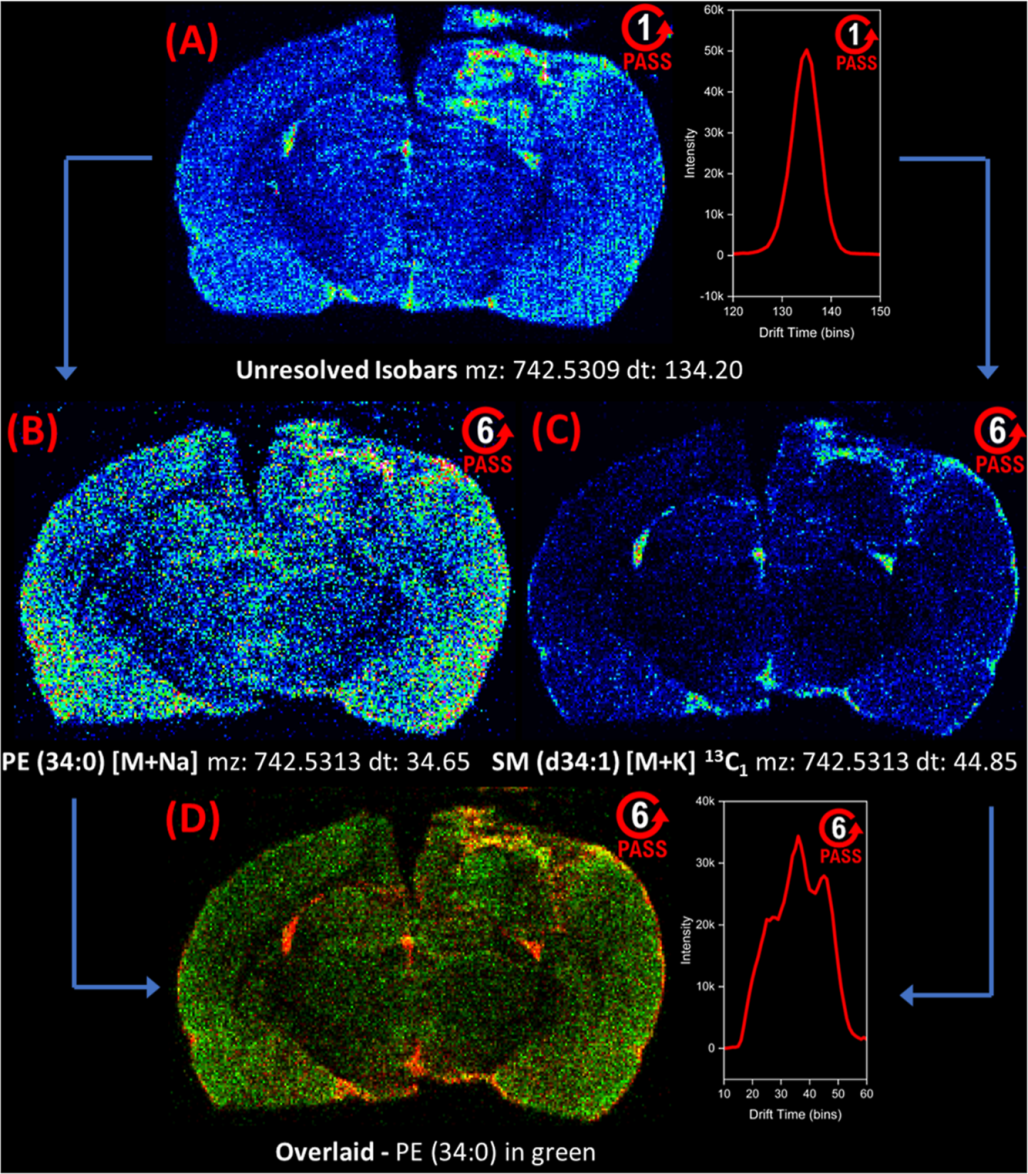
Separating unresolved isobars in a six-pass cyclic mobility experiment. (A) Unresolved ion image from one-pass cIM data that contains two different ion distributions. Fully overlapped isobaric peaks can be seen in the one-pass arrival time distribution plot to the right. (B) Six-pass ion image for PE(34:0) [M + Na]^+^ (*t*_d_ = 34.65 bins), showing its presence throughout the gray matter. (C) Ion image for SM(d34:1) [M + K]^+ 13^C_1_ (*t*_d_ = 44.85 bins) from six-pass data, showing its localization to specific regions of the brain, primarily near the hippocampus, hypothalamus, and the lesion region. (D) An overlaid image of the PE in green and the SM in red, highlights their different distributions. The resolved isobars signals can be seen in the six-pass arrival time distribution plot. See Figure S2 for H&E images of rat 5.

An ion image resembling two distinct distributions was identified in the single pass data at *m*/*z* 742.5309 (Figure 3A). The one pass arrival time distribution plot had a broad peak centered at a *t*_d_ of 134.20 bins, indicating that there were at least two abundant isobaric ions comprising this signal. In the six pass data there were two distinct images at *m*/*z* 742.5313 with *t*_d_ of 34.65 and 44.85 bins (Figure 3B,C). The lower *t*_d_ signal was identified as PE(34:0) [M + Na]^+^ and the higher *t*_d_ signal was identified as SM(d34:1) [M + K]^+ 13^C_1_ isotope. PE(34:0) was distributed throughout the gray matter, while SM(d34:1) was concentrated in very specific regions of the brain, primarily near the hippocampus, hypothalamus, and the lesion area. Differences in the distributions of these species are clearly observed in the overlaid image (Figure 3D). The SM(d34:1) specific signal in red stands out from the much more homogeneous distribution of PE(34:0). Figure S2 shows H&E images of this brain (rat 5) that highlight the impact area and lesion. Subjecting ions to six passes through the cyclic IM cell allowed for separation and individual imaging of isobaric SM and PE, which have similar structures and could not be separated with a single IM pass. The application of cIM in this untargeted MSI study improved specificity and prevented incorrect annotations.

An *m*/*z* 780.5664 ion image showing a unique spatial overlap with the injury site was observed in the single pass DESI cIM-MS data (Figure S4). The corresponding arrival time distribution trace displayed two largely overlapping peaks, indicating that the ion image likely had contributions from two isobaric ions. This mixed signal was not suitable for accurate analysis. However, these isobars were resolved in the six-pass data and could be individually imaged, enabling their specific localization. The first resolved isobar had an *m*/*z* of 780.5641 and a *t*_d_ of 54.17 bins, while isobar 2 had an *m*/*z* of 780.5657 and a *t*_d_ of 71.25 bins. The first isobar was present throughout the gray matter structures outside of the lesion area, while the second was present throughout the white matter and protruded into the lesion area itself. Overlaid images for the resolved isobars showed that they had complementary (and opposite) ion distributions that together resembled the unresolved single-pass image. The presence of isobaric ions at *m*/*z* 780.5664 resulted in an unresolved one-pass ion image that did not reflect the true distribution of the underlying isobars, however cIM allowed these isobaric ions to be individually imaged and their relative abundances plotted.

**Figure 4:**
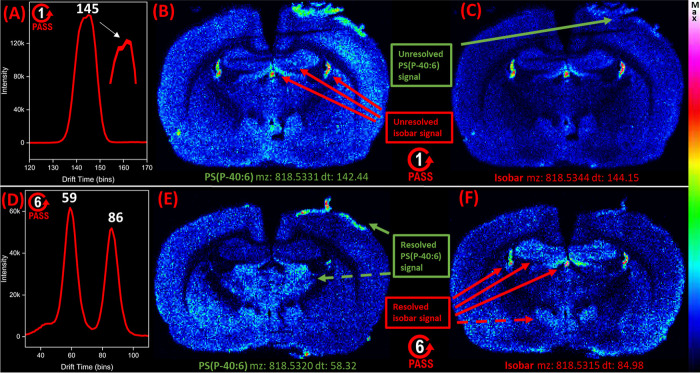
Separating PS(P-40:6) from an isobar with six cIM passes. The top image row depicts single-pass cIM data, demonstrating how the PS(P-40:6) signal overlaps with a second isobaric signal. The bottom row of images is from six-pass experiments, demonstrating the separation of PS(P-40:6) from the isobar. (A) Arrival time distribution trace from single-pass data showing two overlapping peaks at *m*/*z* 818.53. (B) Ion image for PS(P-40:6) from single pass data, with the unresolved isobar signal labeled. (C) Single-pass ion image for the isobar with the unresolved PS(P-40:6) signal labeled at the top of the cortex. (D) Arrival time distribution plot from six-pass data showing that the PS(P-40:6) ion at a *t*_d_ of 59 bins is separated from the isobar at *t*_d_ = 86 bins. (E) Ion image for PS(P-40:6) from six-pass data showing the PS(P-40:6)-specific signal, without the isobar present. The dashed line indicates the area of the brain tissue slice where the PS(P-40:6) signal increased from one-pass to six-pass data. (F) Six-pass ion image showing isobar-specific signals in the vicinity of the hippocampus. The dashed line indicates an area of the brain where the isobar relative signal increased from one-pass to six-pass data. See Figure S2 for H&E images of rat 2.

PS(P-40:6) at *m*/*z* 818.5331 and an isobaric species at *m*/*z* 818.5344 overlapped in one-pass data with a *t*_d_ of 142.44 and 144.15 bins but were separated in six-pass data with a *t*_d_ of 58.32 and 84.98 bins (Figure 4). PS(P-40:6) showed a strong signal around the inflamed tissue near the craniectomy site, while the isobar was primarily concentrated around the hippocampus (Figure 4B,C). However, the PS(P-40:6) signal is present in the isobar one pass image and the isobar signal is present in the PS(P-40:6) one pass image (Figure 4B,C). In the six-pass images, PS(P-40:6) and isobar signals were fully resolved (Figure 4D), and their images showed separate spatial distributions (Figure 4E,F). When comparing the six-pass PS(P-40:6) image to its corresponding one-pass image, the relative signal was stronger in the thalamus, indicated with a dashed arrow. When comparing the six-pass isobar image to its one-pass image, the signal was stronger in the hypothalamus, also indicated with a dashed arrow. This example not only underscores the complexity of the brain lipidome, but also how isobaric lipids can have subtle, yet important differences in their spatial distributions that could be easily overlooked if the experiment does not have adequate specificity, leading to interpretation errors and generation of the wrong hypotheses. cIM helped unravel such complexity by separating isobaric species that were more specifically mapped. Separating these isobars with mass alone would require resolving powers greater than 600,000, which is difficult to achieve even with Fourier Transform Ion Cyclotron Resonance (FTICR) instruments.

### Metabolite Alterations Following Traumatic Brain Injury

Many TBI studies have focused on investigating changes in proteins and complex lipids following injury;^5,6,48,52−55^ however, small metabolites have received considerably less attention despite being important effectors of many central metabolism processes. From the analytical perspective, DESI is ideal for probing small metabolites since it does not require a matrix compound as in matrix-assisted laser desorption/ionization MSI. Such matrices typically result in spectral interferences in the *m*/*z* range below 350 where many metabolites of interest are found.

**Figure 5:**
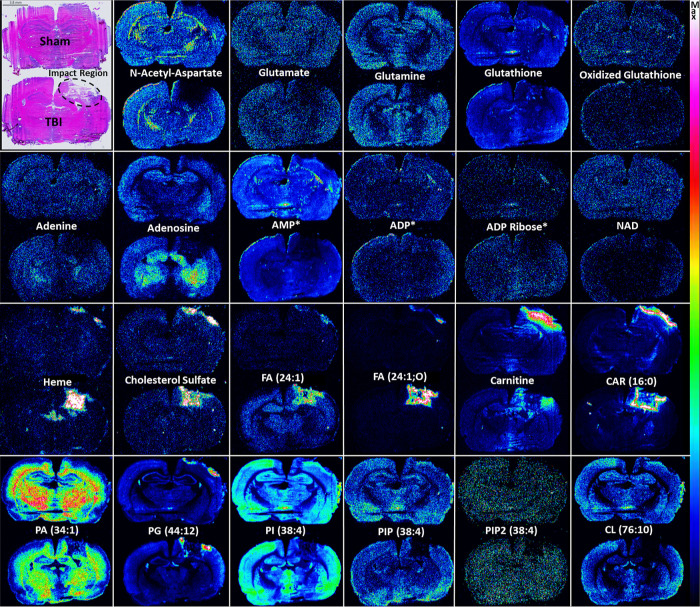
Observed alterations in small molecules and lipids. Representative ion images for various metabolites that were affected by TBI. The top brain tissue section in each image is from a sham rat and the bottom from a TBI rat. The H&E image in the top left corner highlights the impact region in the TBI rat brain. The first row depicts amino acid derivatives, primarily glutamate metabolites. The second row shows adenine metabolites. The third row is for miscellaneous small molecules and lipids. The fourth row shows phospholipids. Putative annotations are provided for each ion image (* = has isomers). See Tables S1 and S2 for additional information on each ion species.

Interesting trends were observed in the brain ion images for many metabolites, despite the modest number of animals included in this pilot study. DESI ion images for select metabolites are shown in Figure 5. Tables S1 and S2 compile the fold change (FC), *p*-value, mass accuracy and *t*_d_ information for metabolites and lipids in the hippocampus ROI contralateral to the craniectomy site, a few of these previously reported in the TBI literature.^5,6,48,52−54^Figure S5 shows the ROI selected for each of the brain tissues under investigation.

Several metabolites depicted in Figure 5 confirmed literature findings linking mitochondrial dysfunction and disrupted energy metabolism with severe TBI.^56,57^ DESI cIM MSI experiments showed carnitine metabolites increased in the hippocampus following injury, while cardiolipins decreased. The carnitine (CAR) shuttle system relies on l-carnitine to transport long-chain fatty acids (FA) into the mitochondria, where they undergo FA oxidation for adenosine triphosphate (ATP) production. The FC of carnitine, CAR(2:0), CAR(16:0), were 1.16, 1.10 and 1.33, respectively. Carnitine increases in the injury area in the vicinity of the surgery region were observed (Figure 5), in agreement with previous reports.^58^Figure S6 depicts the carnitine shuttle pathway and summarizes the related metabolite alterations observed after TBI.

CL decreases associated with injury were observed throughout the brain. CL are four-chained lipids with important structural and functional roles in the carnitine shuttle system and ATP production.^59^ Many mitochondrial oxidative phosphorylation and electron transport reactions, and the stabilization of electron transport chain protein complexes in the mitochondria require CL.^60^ CL decreases have been reported in TBI^53^ and linked to mitochondrial dysfunction^56^ and impaired energy metabolism.^57^ DESI cIM MSI showed significant changes in CL(76:10). This CL was undetectable at the injury site and had a FC of 0.74 in the hippocampus ROI.

Nicotinamide adenine dinucleotide (NAD), an essential cofactor in mitochondrial ATP production,^61^ had the largest decrease in the hippocampus, with an FC of 0.32. NAD also starkly decreased throughout the injured brain (Figure 5). Interestingly, increases in adenosine and decreases in adenosine diphosphate (ADP) appeared to coincide with the region where NAD decreased (Figure 5). Alterations in adenine and some of its metabolites such as adenosine monophosphate (AMP) and ADP-ribose were also detected (Figure 5). The most substantial increases in the hippocampus were for ADP-ribose, deoxycytidine diphosphate (dCDP) and inosine, which had FC of 1.51, 1.41, and 1.37, respectively. dCDP had a relatively low *p*-value of 0.0773. The substantial decrease in NAD, together with CL and nucleotide decreases, further reinforced the idea that energy metabolism in the brain was severely disrupted.^56,57,61^

The lowest *m*/*z* [M – 2H]^2–^ species detected were putatively assigned to phosphoinositides based on accurate mass measurements (PIP, Figure S1). These are phosphorylated PI involved in the recruitment of membrane proteins.^62^ PIP can contain up to three phosphate groups on the 3, 4, and 5 hydroxyl positions of the inositol ring (PIP, PIP2 and PIP3, respectively). Phosphoinositide 3-kinases (PI3K), a family of intracellular enzymes that phosphorylate the 3 position hydroxyl group of the PI inositol ring, have been linked to neuroinflammation.^63,64^ However, alterations in PIP abundances following TBI had not been directly imaged in tissue. DESI cIM MS results showed that PI(38:4), PIP(38:4) and PIP2(38:4) all decreased in the hippocampus, with FC of 0.93, 0.76, and 0.83, respectively (Table S2). Images for these ions are shown in Figure 5. Interestingly, decreases in PIP2 are believed to result in impaired potassium channel function, as PIP2 is required for inward rectifier channels that control blood flow.^65^ To confirm the annotation of the species tentatively assigned to PIP in the brain, chemical standards were spotted and subject to DESI MSI (Figure S7). The PIP(38:4) [M – 2H]^2–^ image showed that the ions in the standard and the brain were identical. PIP(38:4) [M – 2H]^2–^ MS/MS spectra collected post IM confirmed the proposed annotation (Figure S8). Observed inositol bisphosphate fragments were indicative of PIP,^66,67^ with the FA(18:0) and FA(20:4) fragment ions corresponding to the FA chains in PIP(38:4). LC–MS/MS analysis also confirmed that PI(38:4) had the same FA chains (18:0/20:4). We speculated that the detected PIP2(38:4) ion had the same fatty acid chain composition as PI(18:0/20:4) and PIP(18:0/20:4), since brain lipids commonly contain arachidonic acid (AA).^48,68^

PIP decreases in TBI can be attributed to an increased activity of phospholipase A (PLA2) and phospholipase C (PLC) after TBI.^65,69,70^ PLA2 cleaves AA at the PIP(38:4) sn_2_ position leading to increases in free AA. An increase in AA (FA(20:4)) was detected in the hippocampus contralateral to the injury (Figure S9). AA is converted into eicosanoids including prostaglandins and leukotrienes with important pro-neuroinflammatory and antineuroinflammatory roles, respectively.^71^ Increased PLC activity also leads to a decrease in PIP and an increase in diacylglycerols (DG).^65,69,70^ Accordingly, DG(38:4) [M + Na]^+^ and DG(38:4) [M + K]^+^ were found to be highly increased in the injury area (Figure S9). Furthermore, DG(38:4) [M + Na]^+^ had a FC of 1.77 in the contralateral hippocampus with a *p*-value of 0.0312.

The relative phosphorylation extents of PIP were altered by injury. The PIP2(38:4)/PIP(38:4) ratio was slightly higher in TBI than sham brain tissues, with the ratio of TBI/control being 1.16. This indicated that PIP3 phosphatases^72^ and PIP kinases^72^ were activated following injury. In contrast, the PIP(38:4)/PI(38:4) ratio was slightly lower in TBI, implying that PIP2 phosphatases^72^ and PI kinases^72^ were less active following injury. Figure S10 summarizes the PIP metabolic pathway and alterations after TBI.

## Conclusions

This work demonstrated the promise and utility of DESI cIM nontargeted MSI spatial metabolomics experiments for TBI research. Cyclic IM separations, in combination with high resolution mass spectrometry, enabled the separation of numerous isobaric lipids. Without such IM dimension, a blended image of the isobaric species would have been obtained, which would not accurately portray the correct spatial distribution of the detected ions. Higher IM resolving power achieved with six cIM passes allowed for improved separations. An additional advantage of implementing IM in an MSI workflow included the ability to separate groups of multiply charged ions from singly charged ones, and lipids from other biomolecular classes within *t*_d_ vs *m*/*z* plots. This feature was critical in identifying alterations in multiply charged PIP and PIP2 species that have not been previously reported in TBI MSI studies. These decreases are attributed to increase in PLA2 activity after TBI that release AA, which is converted to eicosanoids that control inflammation. DESI allowed the detection of a substantial reduction in NAD and alterations in other adenine metabolites, supporting the hypothesis that energy metabolism in the brain is severely disrupted by TBI.

## Supporting Information Available

Figure S1 shows trend line analysis using drift time vs *m*/*z* plots. Figure S2 shows H&E images for rats used in mass spectrometry images. Figure S3 has additional examples of cardiolipin isobar separation. Figure S4 shows isobar separation in six pass data. Figure S5 shows how the hippocampus ROIs were outlined for fold change analysis. Figure S6 summarizes metabolite changes related to ATP production. Figure S7 shows images collected from phosphoinositide standards and brain. Figure S8 shows MS2 spectra of phosphoinositide standards and brain. Figure S9 shows arachidonic acid containing lipid ion images. Figure S10 summarizes PIP metabolite alterations. (PDF)
(TXT)
