# Supplementary material for: Desorption Electrospray Ionization Cyclic Ion Mobility-Mass Spectrometry Imaging for Traumatic Brain Injury Spatial Metabolomics
Source: Anal Chem. 2024 Aug 6;96(33):13598–606. doi: 10.1021/acs.analchem.4c02394 (PMC11339727; doi:10.1021/acs.analchem.4c02394)
Supplement: Supplementary file 1 — ac4c02394_si_001.pdf [file ac4c02394_si_001.pdf]

Supplemental Information for:

Desorption Electrospray Ionization Cyclic Ion Mobility-Mass Spectrometry Imaging for Traumatic Brain Injury Spatial Metabolomics.

Dmitry Leontyev[1], Hernando Olivos[2], Bindesh Shrestha[2], Pooja M. Datta Roy[3], Michelle C. LaPlaca[3,4*], Facundo M. Fernández[1,4*]

[1]School of Chemistry and Biochemistry, Georgia Institute of Technology, Atlanta, GA 30332 (USA).

[2]Waters Corporation, Milford, MA 01757 (USA).

[3]Coulter Department of Biomedical Engineering, Georgia Institute of Technology/Emory University, Atlanta, GA 30332 (USA).

[4]Parker H. Petit Institute for Bioengineering and Bioscience, Atlanta, GA 30332 (USA).

*Email: michelle.laplaca@bme.gatech.edu
*Email: facundo.fernandez@chemistry.gatech.edu

Supplemental Methods and Materials

Animals and Controlled Cortical Impact Procedures

All animal procedures were conducted in accordance with the guidelines set forth in the Guide for the Care and Use of Laboratory Animals (U.S. Department of Health and Human Services, Washington, DC, USA, Pub no. 85-23, 1985) and approved by the Georgia Institute of Technology Institutional Animal Care and Use Committee (protocol #A100188). To induce open head TBI, male Sprague-Dawley rats (Charles River, Wilmington, MA, USA) weighing between 300-400 grams were kept under 12 h reverse light-dark cycles with food and water given *ad libitum*. Rats were randomly assigned to the sham group (*n*=4) or the injury group (*n*=4). Prior to injury, rats were anesthetized with a cocktail of ketamine/dexmedetomidine (20:0.13 mg kg^{-1}) administered intramuscularly. Once a negative toe pinch reflex was achieved to ensure sedation, rats were secured on a stereotaxic frame, the head shaved, and the area of incision was cleaned with betadine. Aseptic protocols were followed to decrease the chances of contamination and infections. A midline incision was made on the scalp to expose the skull. A craniectomy was made on the right hemisphere tangential to the sagittal and coronal sutures near bregma (+3 mm, +3 mm) using a 5 mm ID/6 mm OD drill bit. (Osada Electric Co. LTD). Once the bone flap was removed, the rat was positioned under a controlled cortical impact (CCI) device. The CCI piston was positioned at a 15° angle. A single impact was administered to the intact dura at a velocity of 5 m s^{-1} and a displacement of 2 mm. For the sham group, craniectomies were performed and animals were placed under the CCI device but did not receive any impacts. The midline incision was closed using 5-6 staples. Rats were then removed from the stereotaxic frame and placed on a heating pad until ambulatory behavior was observed. All animals received injections of antesedan (20 mg kg^{-1}) to reverse the anesthesia and ketoprofen (5 mg kg^{-1}) as an analgesic. Rats were returned to their home cages with special post-surgical bedding. Three days post injury, animals were transcardialy perfused with cold 0.1 M phosphate buffer (pH 7.4).

Liquid Chromatography Mass Spectrometry

Reversed phase ultrahigh-performance liquid chromatography tandem mass spectrometry was collected on brain section homogenates to improve the confidence of lipid annotation following database *m/z* searches and putative annotations. The reversed phase LC MS/MS method used has been previously described in detail by our group[56]. High-energy collision dissociation

(HCD) and collision induced dissociation (CID) data were collected in an Orbitrap ID-X Tribrid mass spectrometer (ThermoFisher Scientific). Chromatography was conducted with a Thermo Accucore C30, 150 × 2.1 mm, 2.6 µm particle size column. Mobile phase A was 10 mM ammonium acetate 0.1 % formic acid in 40:60 v/v water/acetonitrile. Mobile phase B was 10 mM ammonium acetate 0.1 % formic acid in 90:10 v/v isopropanol/acetonitrile.

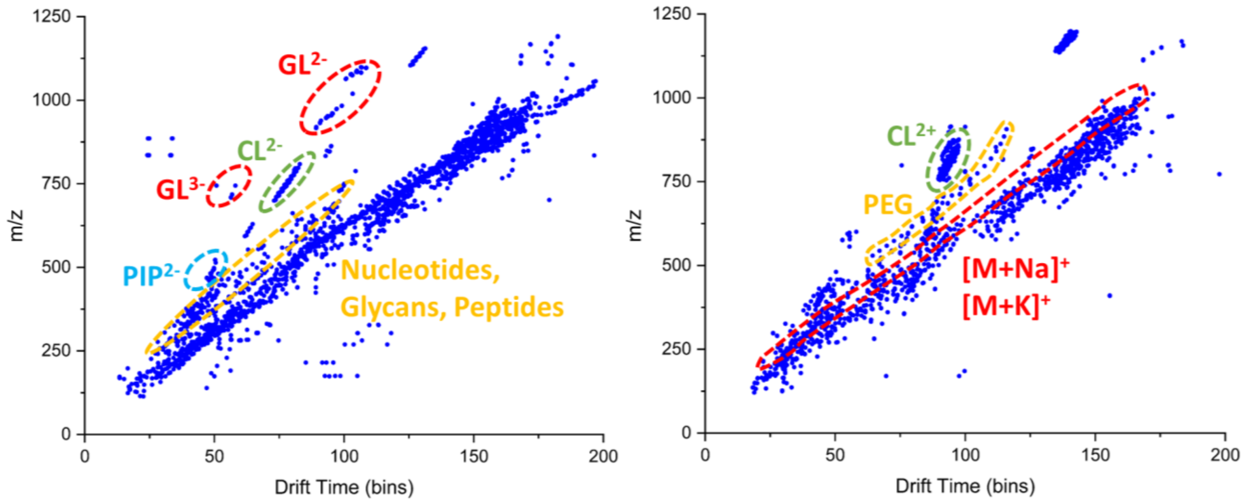

Figure S1. Trend line analysis using drift time (t_d) *vs. m/z* plots. Drift time (t_d) plots for negative (left) and positive (right) ion modes using the 1000 most intense peaks. Compound class identity for a number of ion groups is provided. The most salient ion classes that were distinguishable from the singly deprotonated lipids in negative ion mode were the singly deprotonated nucleotides, glycans and peptides, doubly deprotonated phosphoinositides (PIP), cardiolipins (CL), and gangliosides (GL), and triply deprotonated GL. The most salient ion classes separated by t_d from the singly protonated lipids in positive ion mode were sodium and potassium lipids adducts, doubly protonated CL and polyethylene glycol species (PEG). 2+ = $[M+2H]^{2+}$, 2- = $[M-2H]^{2-}$, 3- = [M-3H]$^{3-}$.

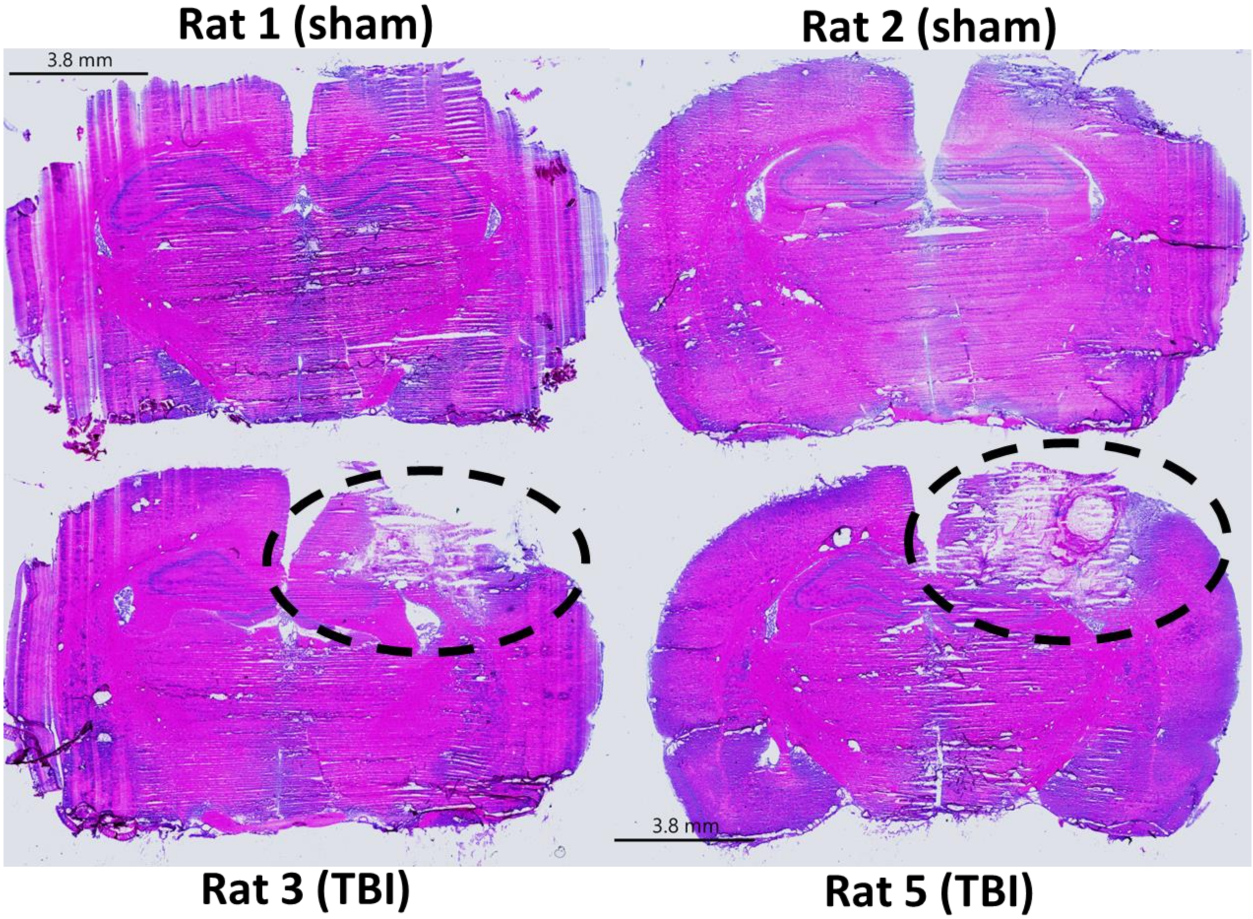

Figure S2. H&E images of rat brains to highlight impact area. Rat 1 and 2 are sham, whereas rat 3 and 5 received a TBI. The right side of rat 3 and 5 highlight the lesion. Figure 2 in the manuscript used rat 3 images, Figure 3 used rat 5 images and Figure 4 used rat 2 images.

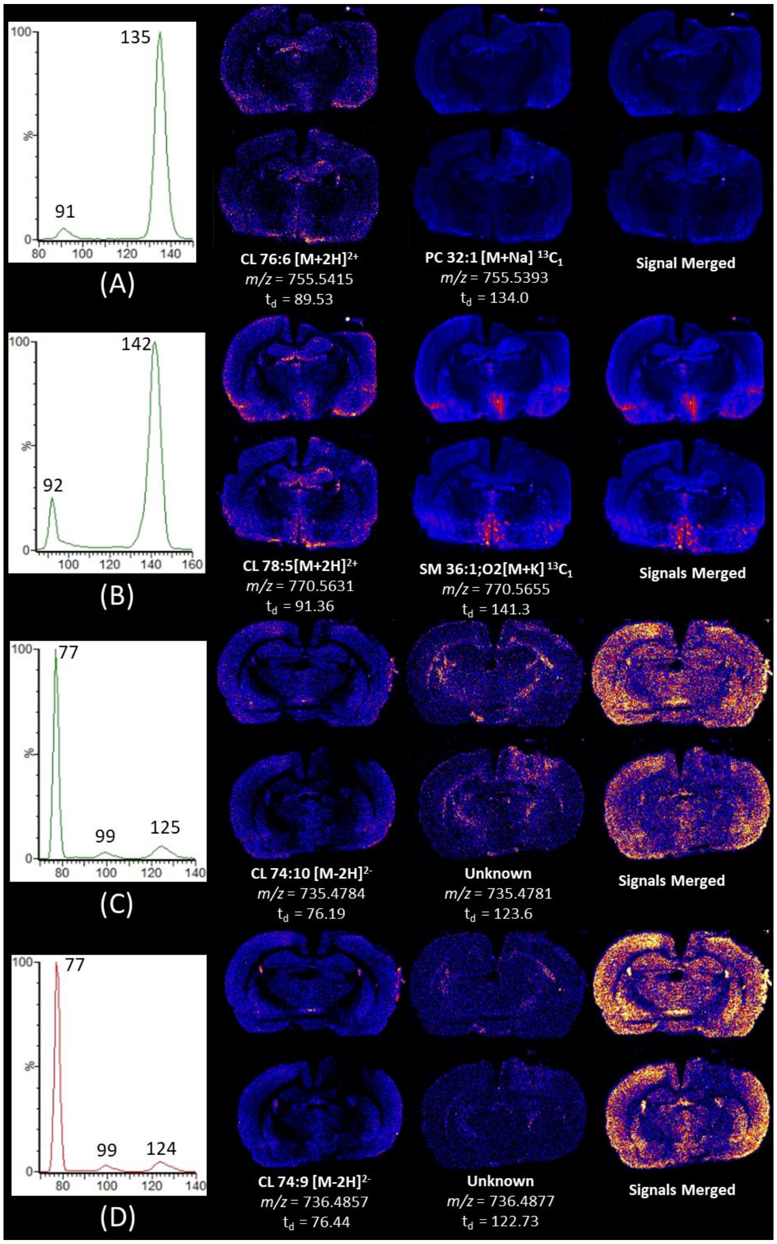

Figure S3. Separating doubly charged cardiolipins from singly charged isobars with one pass of cyclic ion mobility experiments. **(A)** Arrival time distribution trace showing two signals at *m/z* 755.54. The lower drift time signal is CL(76:6). When merged with the ^{13}C isotope of PC(32:1) $[M+Na]^{+}$, the CL signal is overpowered by the PC. **(B)** Arrival time distribution trace showing two signals at *m/z* 770.56. The lower drift time signal is CL(78:5), when merged with SM(d36:1) $[M+Na]^{+}$, the CL signal is overpowered by the SM. **(C)** Arrival time distribution trace showing three distinct signals at *m/z* 735.47. The lowest drift time signal is CL(74:10). When merged with the isobaric signal at a 123.6 drift time, the CL signal is obscured by the isobar. **(D)** Arrival time distribution trace at *m/z* 736.48. The lowest drift time signal is CL(74:9). When merged with the isobaric signal at 122.73 drift time, the CL signal is severely obscured.

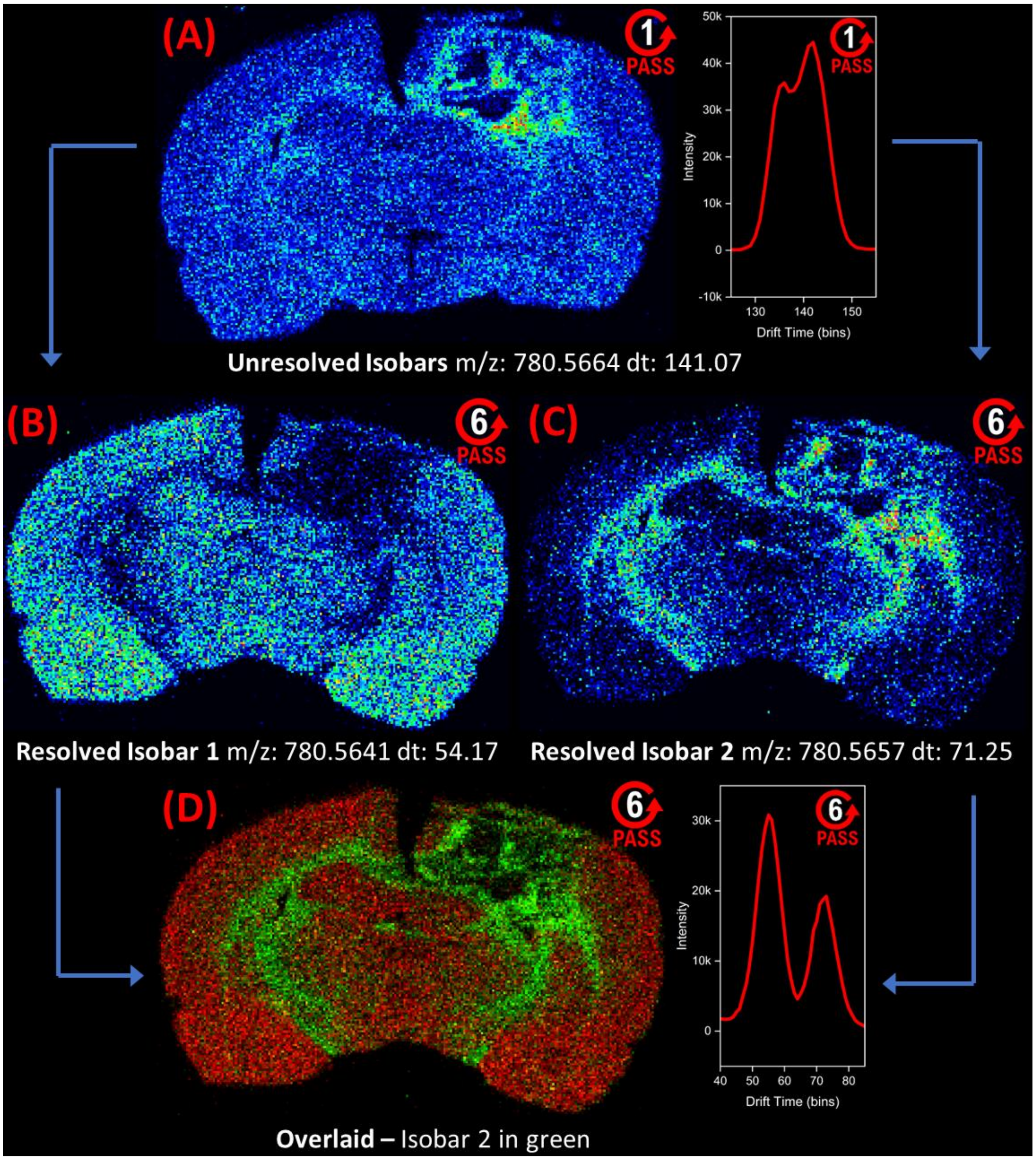

Figure S4. Separating unresolved isobars in a six-pass cyclic mobility experiment. **(A)** Ion image at *m/z* 780.5664 from one-pass cIM data showing a marked increase at the injury site. Overlapping isobaric peaks can be seen in the one-pass arrival time distribution trace to the right. **(B)** Ion image for the first resolved isobar (t_d= 54.17 bins) from six-pass data, showing it present throughout the gray matter. **(C)** Ion image for the second resolved isobar observed in six-pass data (t_d=71.25 bins), showing its presence throughout the white matter and the lesion area. **(D)** The overlaid image of the first isobar in red and the second in green, showing their spatially-complementary distributions, and the resemblance of the merged ion image to the original unresolved one-pass image. The resolved isobars signals can be seen in the six-pass arrival time distribution plot.

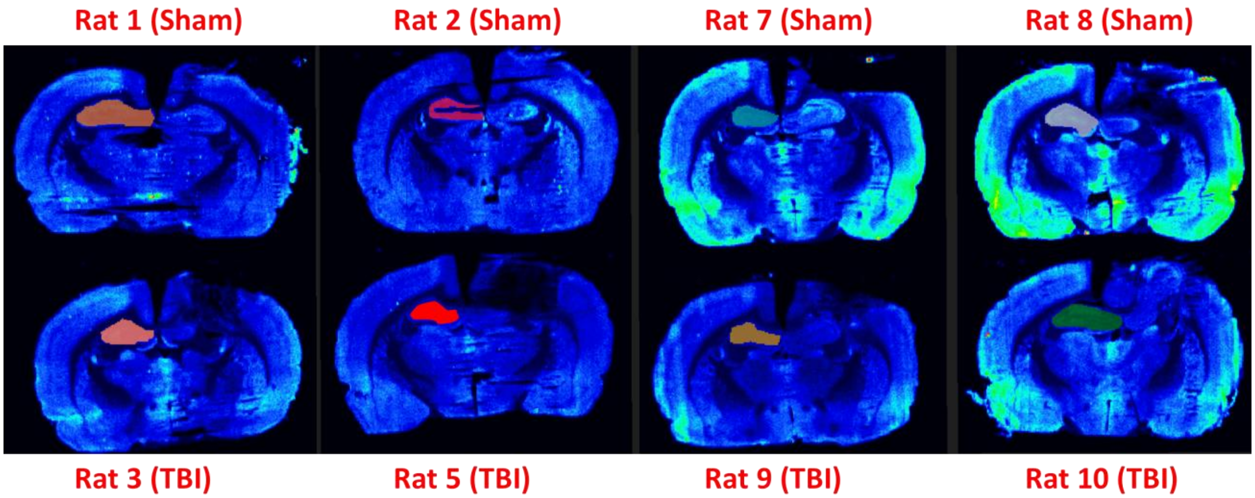

Figure S5. Hippocampus region of interest selection. Ion images of PI (38:4) for all rat brains. Shown is the selection of the hippocampus for all 8 rats in negative ion mode to estimate abundance fold changes.

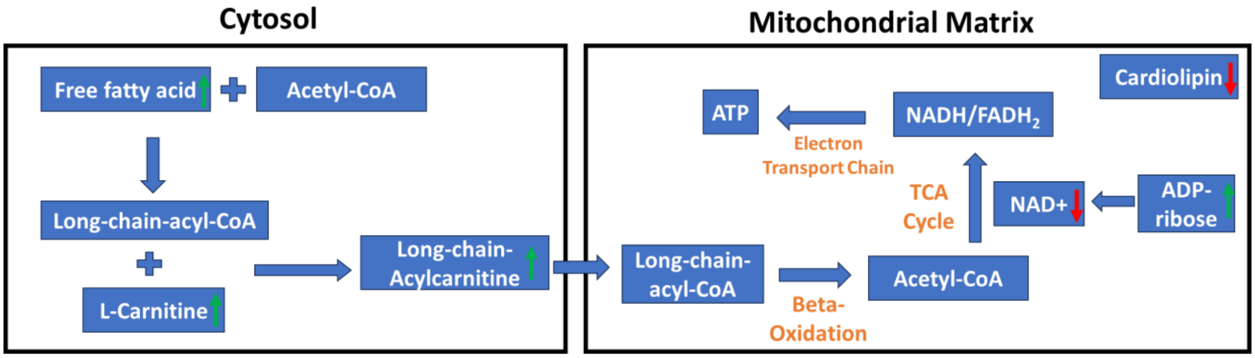

Figure S6. Carnitine shuttle and beta oxidation pathway alterations after TBI. Increases in free fatty acid, L-carnitine, long-chain-acylcarnitines and ADP-ribose were observed following TBI. Decreases in cardiolipin and NAD were also observed.

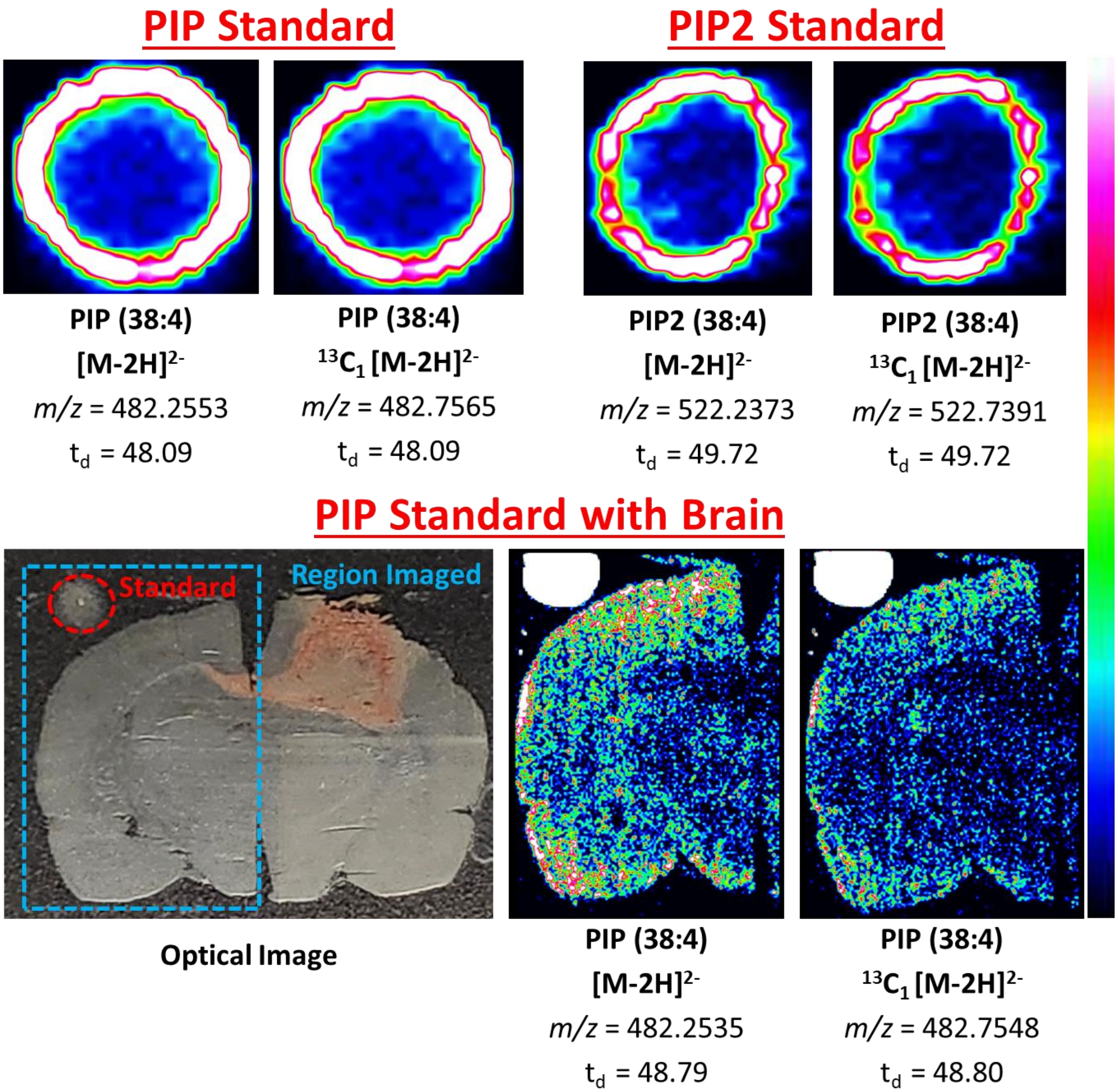

Figure S7. Phosphoinositide ion images. A naturally derived PIP mixture and PIP2 mixture were spotted onto slides and imaged. Ion images are shown for PIP (38:4), PIP2 (38:4), and their $^{13}C_1$ isotope. The PIP standard mixture was spotted next to a brain and imaged together. Ion images are shown for PIP (38:4) and its $^{13}C_1$ isotope. The standard and brain shared the same PIP (38:4) signals at the same *m/z* and drift time. The *m/z* difference between isotopic signals indicated these were doubly charged species. These species had lower drift time (~50 bin) than the average singly deprotonated ion at a similar *m/z* (~75 bin).

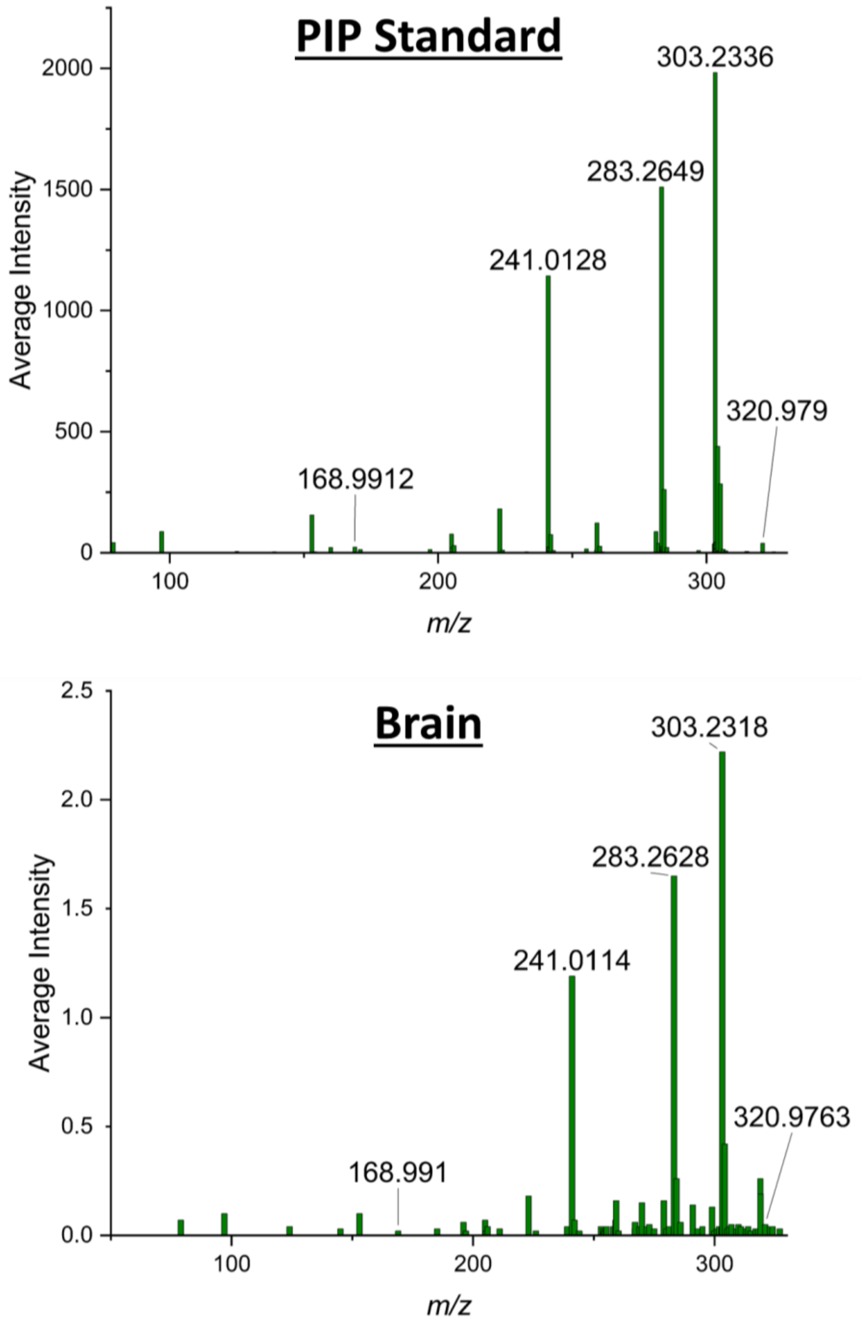

Figure S8. MS/MS spectra for phosphoinostide. Comparison of the MS2 spectra for the PIP (38:4) $[M-2H]^{2-}$ ion at *m/z* 482.2 collected for a PIP standard (top) and brain tissue (bottom). The inositol bisphosphate fragments are diagnostic for PIP. All labeled fragments had drift times between 46.80 and 48.31 bins, which are in line with the drift time of PIP (38:4). *m/z* 168.99 = Inositol bisphosphate $[M-2H]^{2-}$; *m/z* 241.01 = Inositol phosphate $[M-H_2O-H]^-$; *m/z* 283.26 = FA (18:0) $[M-H]^-$; *m/z* 303.23 = FA (20:4) $[M-H]^-$; *m/z* 320.97 = Inositol bisphosphate $[M-H_2O-H]^-$.

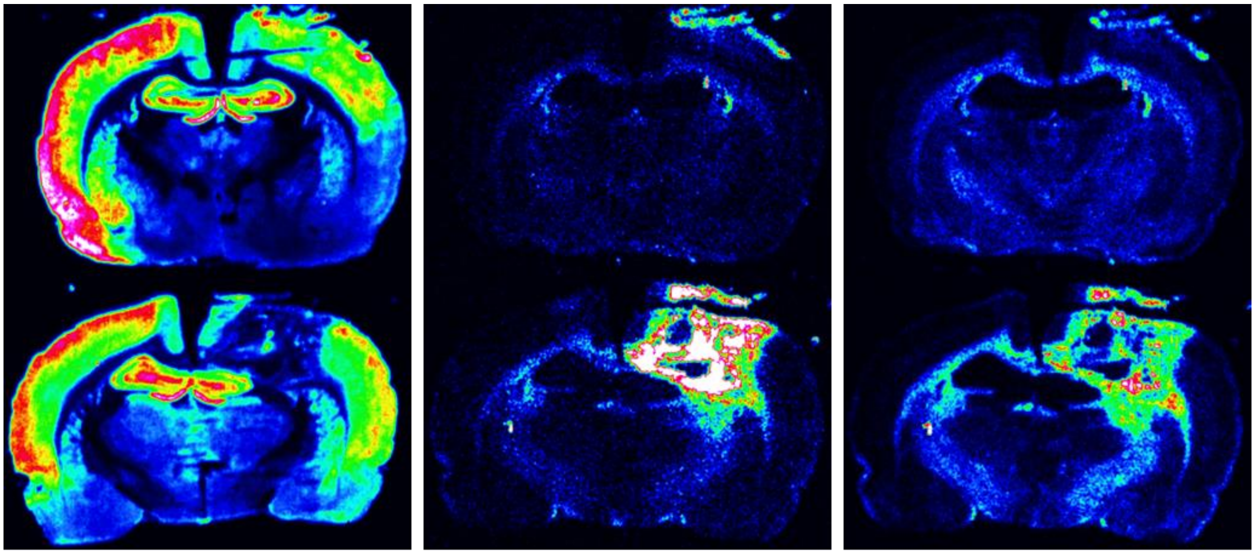

FA(20:4) $[M-H]^-$ | DG(38:4) $[M+Na]^+$ | DG(38:4) $[M+K]^+$

m/z = 303.2332 | m/z = 667.5248 | m/z = 683.5000

t_d = 51.73 | t_d = 120.42 | t_d = 120.30

Figure S9. Ion images for arachidonic acid-containing metabolites. The FA(20:4) ion image shows an increase in the contralateral hippocampus. The DG(38:4) ion images demonstrate an increase in the lesion area.

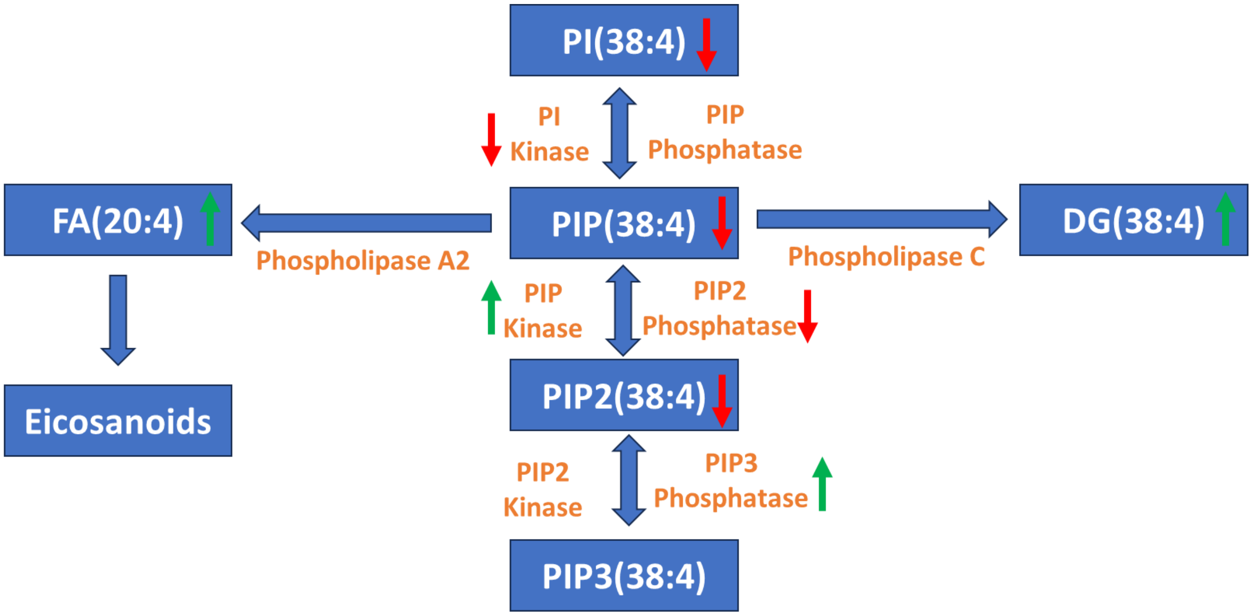

Figure S10. PIP metabolic pathway and alterations after TBI. PI(38:4), PIP(38:4) and PIP2(38:4) all decreased after TBI. Arachidonic acid (FA(20:4)) and DG(38:4) increased after TBI, reflecting increases in Phospholipase A2 and Phospholipase C, respectively. PIP2(38:4)/PIP(38:4) ratio was slightly higher in TBI than sham brain tissues, indicating that PIP3 phosphatases and PIP kinases were activated after TBI. In contrast, the PIP(38:4)/PI(38:4) ratio was slightly lower in TBI, implying that PIP2 phosphatases and PI kinases were less active following injury.

Table S1. Small molecule alterations following TBI. Data for selected metabolites, 28 detected in negative ion mode and 7 in positive ion mode. Fold change (FC) was calculated from the hippocampus (HC) region contralateral to the craniectomy as the ratio of injured/control (>1 means increased in injured). P-values were calculated from a two sample t-test. Acronyms were used for nucleotides, see HMDB for full name. All ions are $[M-H]^-$ unless specified. $^H = [M+H]^+$, $^K = [M+K]^+$, * = has isomers.

Small Molecule ID	HC FC	P-value	*m/z*	Mass Error (ppm)	t_d (bins)
Fumurate	1.04	0.7060	115.0033	-3.3	21.36
Creatine^H	1.04	0.8849	132.0765	-1.9	19.44
Adenine	1.04	0.8493	134.0470	-1.6	17.50
Hypoxanthine	1.33	**0.1050**	135.0313	0.7	17.25
Glutamate	0.97	0.7922	146.0456	-1.9	20.27
Carnitine^H	1.16	0.5115	162.1122	-1.7	23.68
N-Acetyl-Aspartic-Acid	0.93	0.4231	174.0406	0.0	22.88
Ascorbic acid	1.05	0.6859	175.0246	-1.2	21.56
Glutamine^K	1.13	0.5821	185.0321	-1.1	99.73
Glycerylphosphorylethanolamine	0.92	0.4743	214.0486	0.0	28.29
Glycerophosphoglycerol	0.98	0.9119	245.0432	0.4	31.24
Aspartyl-4-phosphate^K	0.80	0.2530	251.9668	-0.8	32.83
Myo-Inositol 6-phosphate*	1.01	0.9090	259.0223	-0.6	30.28
Inosine	1.37	0.2415	267.0737	0.8	35.70
Adenosine^H	1.22	0.2504	268.1035	-0.5	34.32
Glycerophosphocholine^K	0.70	**0.1078**	296.0654	-2.0	37.63
Glutathione	1.12	0.4390	306.0766	0.2	38.88
CMP	1.04	0.8515	322.0446	0.7	39.51
UMP*	0.98	0.9201	323.0287	0.3	38.41
Glycerophosphoinositol	0.98	0.8246	333.0589	-0.9	40.45
Fructose 1,6-bisphosphate*	1.01	0.9713	338.9882	-1.8	35.81
AMP*	0.92	0.7805	346.0561	0.8	44.10
IMP	0.99	0.9433	347.0413	4.3	43.28
GMP	1.03	0.7268	362.0507	-0.1	41.96
Phosphatidylserine	0.85	0.2421	384.1060	-1.3	51.27
dCDP	1.41	**0.0773**	386.0173	3.4	47.82
ADP*	0.89	0.7530	426.0224	0.7	51.40
CDP-ethanolamine	0.92	0.5675	445.0532	0.2	54.12
Cholesterol Sulfate	1.14	**0.1646**	465.3055	2.4	89.13
ADP Ribose*	1.51	**0.1381**	558.0648	0.7	68.23
UDP-Glucose*	0.98	0.9425	565.0480	0.5	69.00
UDP-N-Acetylglucosamine	0.92	0.7250	606.0746	0.5	79.88
Oxidized Glutathione	0.37	**0.0126**	611.145	0.5	76.16
Heme^H	0.94	0.8034	616.1759	-2.3	92.64
NAD	0.32	**0.2011**	662.1008	-1.5	80.14

Table S2. Lipid alterations following TBI. Data for selected metabolites, 30 detected in negative ion mode and 5 in positive ion mode. Fold change (FC) was calculated from the hippocampus (HC) region contralateral to the craniectomy as the ratio of injured/control (>1 means increased in injured). P-values were calculated from a two sample t-test. All ions are $[M\text{-}H]^-$ unless specified. ^{H} = $[M+H]^+$, ^{Na} = $[M+Na]^+$, ^{K} = $[M+K]^+$, $^{2H-}$ = $[M\text{-}2H]^{2-}$, * = has isomers.

Lipid ID	HC FC	P-value	*m/z*	Mass Error (ppm)	t_d (bins)
CAR 2:0^{H}	1.10	0.7816	204.1227	-1.5	29.08
FA 20:4	1.09	0.4466	303.2332	0.7	51.73
FA 24:1	1.36	0.4809	365.3430	1.4	62.79
FA 24:1;O	1.22	0.3978	381.3375	0.3	65.19
CAR 16:0^{H}	1.33	0.3127	400.3411	-2.5	73.52
LPE O-18:1	0.97	0.5247	464.3154	1.5	76.33
LPS O-16:1	0.77	**0.0002**	480.2728	-0.8	76.10
PIP 38:4^{2H-}	0.76	0.4292	482.2539	-1.2	48.81
PIP2 38:4^{2H-}	0.83	0.4401	522.2372	-0.8	50.26
PA O-34:1	0.80	0.1518	659.5013	-1.2	112.73
DG 38:4^{Na}	1.77	**0.0312**	667.5248	-3.6	120.42
PA 34:1	0.81	0.2331	673.4822	1.2	113.85
DG 38:4^{K}	1.04	0.9031	683.5000	-1.6	120.30
PA O-36:2	0.85	0.3726	685.5175	-0.4	118.26
PA 36:2	0.70	**0.1446**	699.4978	1.1	117.9
SM d36:1^{H}	0.96	0.2930	731.6048	-1.8	139.00
PE 36:1	1.04	0.6063	744.5543	-0.8	126.71
PE O-38:7	0.95	**0.1898**	746.5136	0.8	126.46
CL 76:10^{2H-}	0.74	**0.0952**	749.4927	-2.4	77.84
PE 38:6	0.98	0.7342	762.5073	-0.8	127.35
PE O-40:7	1.00	0.9456	774.5444	0.1	132.25
PC 36:1^{H}	1.07	0.4920	788.6148	-2.0	143.74
PE 40:6	1.02	0.6837	790.5411	2.4	133.60
PG 38:4	0.91	0.5832	797.5336	-0.3	135.39
PS O-40:7	0.94	0.3345	818.5331	-1.3	142.44
PS 40:7	0.89	**0.1306**	832.5140	0.7	140.37
PS 40:6	0.91	**0.1190**	834.5305	1.7	142.16
PS 40:4	0.92	**0.1076**	838.5605	0.1	144.11
PS O-42:7	1.06	0.4340	846.5637	-2.1	151.41
PI 36:4	0.90	**0.0429**	857.5194	0.9	143.71
PG 44:12	1.06	0.7523	865.5040	1.7	147.33
PI 38:6	0.91	**0.0133**	881.5186	0	147.40
PI 38:4	0.93	**0.1510**	885.5518	2.1	149.78
PI 40:6	0.98	0.7189	909.5510	1.2	153.71
GD1a*$^{2H-}$	1.11	0.6036	917.4794	1.3	89.28
